# Corrigendum to “Early postoperative infection in patient with IgM deficiency”

**DOI:** 10.1111/iwj.70032

**Published:** 2024-08-21

Chan K, Loh CYY. Early postoperative infection in patient with IgM deficiency. *Int Wound J*. 2024;21(7):e70003. doi:10.1111/iwj.70003 PMC1125302339016243

Charles Yuen Yung Loh is the senior author of the Letter and should be added as co‐corresponding author. Any queries or questions from readers can be directed to him. The email address is charles.loh1@nhs.net.

